# The role of visual awareness for conflict adaptation in the masked priming task: comparing block-wise adaptation with trial-by-trial adaptation

**DOI:** 10.3389/fpsyg.2014.01347

**Published:** 2014-11-25

**Authors:** Kunihiro Hasegawa, Shin’ya Takahashi

**Affiliations:** ^1^Department of Psychology, Nagoya UniversityNagoya, Japan; ^2^Department of Psychology, Tokaigakuen UniversityNagoya, Japan

**Keywords:** cognitive control, subliminal priming, conflict adaptation, proportion congruency, contingency learning

## Abstract

This study investigated the role of participants’ visual awareness in the block-wise and the trial-by-trial adaptations. We employed a subliminal response compatibility task in which a prime arrow was briefly presented before the target arrow, and the participants were requested to indicate the direction of the target arrow. The direction of the prime and direction of the target were either the same (compatible trial) or different (incompatible trial). To examine block-wise adaptation, two blocks were conducted, i.e., the Neutral block (50% compatible and 50% incompatible trials) and the Incompatible block (10% compatible and 90% incompatible trials). The results showed the existence of the block-wise adaptation without participants’ visual awareness. The compatibility effect on both response time and error rate (ER) was smaller in the Incompatible block than in the Neutral block. Moreover, a separate data analysis based on the preceding trial type revealed that the trial-by-trial adaptation of cognitive control was observed only in the ER. These results suggest the different role of visual awareness in the block-wise and trial-by-trial adaptations.

## INTRODUCTION

Cognitive control is one of the most important cognitive functions humans have for environmental adaptation. By employing response compatibility tasks in a laboratory setting, we are able to examine the process of selecting an appropriate stimulus and guiding participants to an optimized behavior. For example, in the flanker task (Eriksen and Eriksen, 1974), response time (RT) is generally faster when the central target is surrounded by compatible flankers (e.g.,<<<<<) as opposed to incompatible flankers (e.g.,>><>>). The difference between RTs (RT in the incompatible trials minus RT in the compatible trials) is called the “compatibility effect” and regarded as an index of efficiency of conflict solving (Nieuwenhuis et al., 2006; Verguts and Notebaert, 2009).

Amazingly, the compatibility effect is known to be modulated by the task context. Earlier studies reported a block-wise context effect in the response compatibility task. In a block with a larger number of incompatible trials (e.g., 90% incompatible and 10% compatible), the compatibility effect becomes very small and sometimes changes its direction to yield a reverse compatibility effect (Logan and Zbrodoff, 1979; Lindsay and Jacoby, 1994). Furthermore, a trial-by-trial sequential analysis revealed that the compatibility effect is smaller when the preceding trial type is incompatible vs. compatible (Gratton et al., 1992; Stürmer et al., 2002), while the compatibility effect is clearly observed for all the data in a block.

To completely understand these adaptation mechanisms, recent studies have highlighted two issues: (1) differences between block-wise and trial-by-trial adaptations (Braver, 2012) and (2) the role of the participant’s awareness in these adaptations (Desender and Van den Bussche, 2012; Schmidt and de Houwer, 2012; Desender et al., 2013). In regard to the first issue, our recent study, which investigated the false alarm response in no-go trials in the Simon task, demonstrated the difference between these adaptations in the process of task-irrelevant information (Hasegawa and Takahashi, 2013). In the experiments, a red, green, or gray disk was presented on either the left or right side of a monitor. Participants were requested to respond to a red or green disk by pressing assigned keys while ignoring its location (normal Simon trials) and to refrain from responding when a gray disk was presented (no-go trial). When the trial-by-trial context was examined, the overall rate of the false alarm response (key-pressing for the gray disk) was lower when the no-go trial was immediately preceded by the incompatible trial compared to the compatible trial, suggesting the enhancement of the inhibition of the task-irrelevant process. This result is well explained by the conflict monitoring theory (Botvinick et al., 2001, 2004), which emphasizes that experience of conflict in one trial boosts inhibition of the task-irrelevant response activation in the next trial. More specifically, the trial-by-trial adaptation is explained as a feedback-loop of the conflict detection and the top-down control demand, and when the conflict is detected in the current trial, the top–down control demand in the next trial is assumed to be strengthened.

Conversely, when the block-wise context was manipulated, a utilization of the task-irrelevant information rather than its inhibition was suggested. This was shown by the fact that when a block contained a larger number of incompatible trials, the opposite-side false alarm (i.e., to respond with the right hand to a gray disk presented on the left side and vice versa) occurred more frequently than the same-side false alarm, whereas the overall false alarm rate was not changed by the manipulation of the block-wise context. These incompatible results for the trial-by-trial and the block-wise contexts suggest that the block-wise adaptation is not a simple accumulation of the trial-by-trial adaptation, contrary to a presumption of the conflict monitoring theory. We have argued that the contingency learning model (Schmidt, 2013) would better explain our results of the block-wise adaptation; participants would learn contingency between the correct response and the task-irrelevant location information to make reactive bias of responding with the hand opposite to the stimulus location (Hasegawa and Takahashi, 2013).

Next, as for the second issue, there is no direct evidence supporting the role of awareness for cognitive control in the response conflict task. Though the effect of block-wise adaptation has been often argued to be a conscious control, our abovementioned study suggested that block-wise adaptation is achieved in an unconscious manner (Hasegawa and Takahashi, 2013). It was shown that our participants were not aware of the utilization of the task irrelevant information, and furthermore, they were not aware of the proportion of the trial types (compatible vs. incompatible) that was manipulated in two blocks.

In relation to the trial-by-trial adaptation, there have been some investigations of the role of awareness, but the results are incompatible with each other. For example, Kunde (2003) showed an absence of trial-by-trial adaptation in the unconscious response conflict task. In his study, a task-irrelevant priming arrow was presented for 14 ms before presenting the target arrow, which ensured that the pointing direction of the priming arrow was not discriminable. The results showed that in the incompatible trials (i.e., the priming arrow pointed in the opposite direction of the target arrow), RT was longer and error rate (ER) was larger than in the compatible trials (both arrows pointed to the same direction). Moreover, this compatibility effect was not influenced by a trial sequence, thereby suggesting the necessity of the awareness of conflict for the trial-by-trial adaptation. However, more recent studies using the same task did show the trial-by-trial adaptation of ER (van Gaal et al., 2010; Francken et al., 2011) and RT (van Gaal et al., 2010). These conflicting results (**Table 1**) demonstrate that the role of awareness in trial-by-trial adaptation remains unclear.

**Table 1:** Summary of unconscious trial-by-trial adaptation effects in previous studies.

	RT adaptation		ER adaptation	
Kunde (2003)	–2 ms	n.s.	1.3 %	Not analyzed
van Gaal et al. (2010)	9 ms	**	1.7 %	*
Francken et al. (2011)	1 ms	+	4.1 %	****
				

^+^*p*< 0.10, *p< 0.05, **p< 0.01, ****p< 0.001.

This study aims to reveal the role of visual awareness in the block-wise and trial-by-trial adaptations. Therefore, we employed the subliminal response conflict task (Kunde, 2003), and compared the compatibility effects between the Neutral block (including 50% incompatible and 50% compatible trials) and the Incompatible block (including 90% incompatible and 10% compatible trials). In addition, data were analyzed separately for trials immediately followed the compatible trial and trials immediately followed the incompatible trial to examine the effect of the trial-by-trial adaptation.

## MATERIALS AND METHODS

### PARTICIPANTS

Twenty volunteers (10 females and 10 males, 19–28 years of age, *M* = 22.0) participated. All reported having normal or corrected-to-normal vision. All participants provided written informed consent. They provided permission for their data to be used in the analysis.

### APPARATUS AND STIMULI

The stimuli were displayed on a CRT monitor (Sony GDM-F520) controlled by a computer (Apple MB324J/A) and Psychophysics Toolbox (Brainard, 1997). The monitor refresh rate was 100 Hz.

**FIGURE 1:**
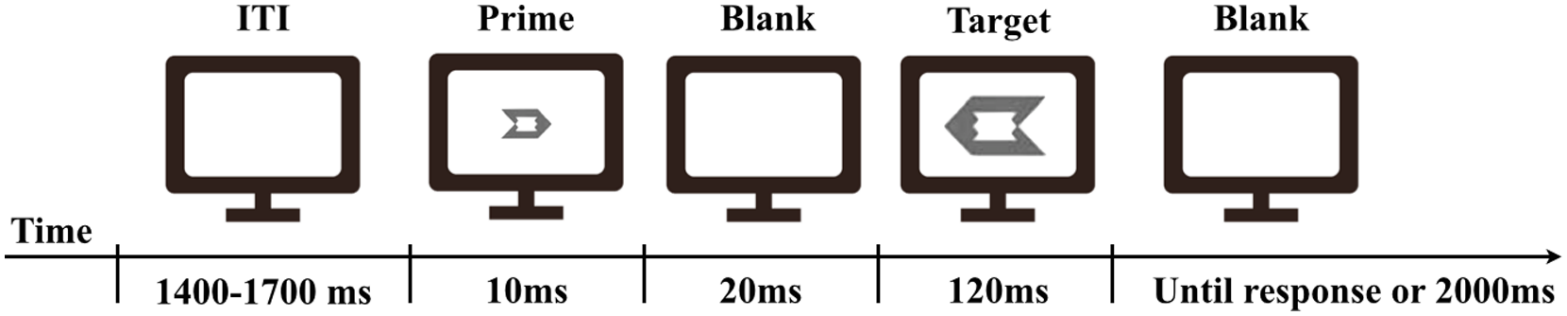
**Timeline of the experimental trial**.

In the masked priming task, a left-pointing or right-pointing arrow-like figure was used as the prime and the target. The prime was 0.7°× 1, and the target was 1.7°× 2.4 of visual angle. The prime fitted exactly into the space in the middle of the target (**Figure 1**). The mask used in the prime discrimination task was depicted as an overlapped figure of the left-pointing and right-pointing targets.

### PROCEDURE

In the masked priming task, the prime was presented for 10 ms, and then, following a blank interval of 20 ms, the target was presented for 120 ms. Participants were requested to press, as quickly and accurately as possible, the “F” key on a keyboard with the left index finger for the left-pointing target, and a “J” key with the right index finger for the right-pointing target. The next trial was started after a response was made or 2000 ms passed without a response. The length of the inter-trial-interval (ITI) was varied randomly within a range of 1400–1700 ms (**Figure 1**).

Participants performed two separate blocks (Neutral and Incompatible), which each consisted of 320 experimental trials. The Neutral block had 160 compatible trials (the left-pointing target followed the left-pointing prime or the right-pointing target followed the right-pointing prime) and 160 incompatible trials (the left-pointing target followed the right-pointing prime or the right-pointing target followed the left-pointing prime). The Incompatible block had 32 compatible trials and 288 incompatible trials. Participants were not provided with any information about the presentation of the prime and the proportion of compatible/incompatible trials in each block. The block order was fixed for all participants. The Neutral block was performed first. This was because the possible biased effect of the Incompatible block should not be carried over into the Neutral block. The trial order in each block was randomized among participants. After completing the second block, they were questioned whether they noticed any difference between the two blocks.

Next, the prime discrimination task was conducted. Before beginning the task, the participants were informed that a prime was briefly presented before the target in each trial of the masked priming task they had just completed. Then, in the prime discrimination task, the prime was presented for 10 ms, and, following a blank interval of 20 ms, the mask was presented for 120 ms. Participants were requested to answer the pointing direction of the prime (a two-alternative forced choice between left and right). Forty trials (20 left and 20 right) were provided in a random order to each participant.

## RESULTS

### PRIME DISCRIMINATION TASK

The mean correct response rate in the prime discrimination task was 51.75%, which was not significantly different from the chance level (50%), *t*(19) = 1.017, *p* = 0.322, *d* = 0.346. This ensures that the pointing direction of the prime was not discriminable and the employed masked priming task worked properly as a subliminal conflict task. Furthermore, this is also supported by the result of the post-task interview concerning the participants’ noticing of any difference between the two blocks; that is, none of them pointed out the difference between the blocks.

### RESPONSE TIME AND ERROR RATE IN THE MASKED PRIMING TASK

In the analysis of RT data, trials that elicited an incorrect response were excluded. In addition, the criterion for the outliers was set at ± 2.5 SD of mean RT in each participant; however, actually, there was no outlier in the whole data.

Mean RT in the masked priming task (**Table 2**) was analyzed by a three-way repeated measures analysis of variance (ANOVA) with the block type (Neutral and Incompatible), the preceding trial type (compatible and incompatible), and the current trial type (compatible and incompatible). There were a significant main effect of the current trial type, *F*(1,19) = 189.06, *p* < 0.001, $\eta_{p}^{2}$ = 0.909, and a significant interaction between the block type and the current trial type, *F*(1,19) = 4.88, *p* = 0.040, $\eta_{p}^{2}$ = 0.204. Any other main effects or interactions were not significant. *Post hoc* analysis indicated that RT on the incompatible current trial was longer than RT on the compatible current trial in each block, *p* < 0.001 in the Neutral block; *p* < 0.001 in the Incompatible block. In addition, RT on the incompatible current trial was significantly shorter in the Incompatible block than in the Neutral block, *p* = 0.012, whereas RT on the compatible current trial was not different between blocks, *p* = 0.645.

**Table 2:** Mean response time (RT) and error rate (ER) in the masked priming task.

		Current Trial			
		Compatible		Incompatible	
		RT (SD)	ER (SD)	RT (SD)	ER (SD)
**Neutral Block (50%-Incompatible)**						
Preceding trial	Compatible	273.5 (37.4)	1.2 (0.0)	335.4 (37.0)	12.7 (0.1)
	Incompatible	269.7 (35.8)	0.8 (0.0)	336.8 (37.1)	8.6 (0.1)
**Incompatible Block (90%-Compatible)**						
Preceding trial	Compatible	266.2 (63.5)	0.0 (0.0)	321.7 (37.9)	5.6 (0.1)
	Incompatible	271.9 (29.6)	0.8 (0.0)	321.5 (30.7)	6.0 (0.1)

*RT, mean reaction time (ms); ER, error rate (%).*

Mean ER in the masked priming task (**Table 2**) was analyzed by the same three-way ANOVA as in the case of RT. As a result, a three-way interaction was significant, *F*(1,19) = 4.801, *p* = 0.041, $\eta_{p}^{2}$ = 0.202. *Post hoc* analyses indicated that ER on the incompatible current trial in the Neutral block was lower when the preceding trial was incompatible than compatible (*p* < 0.001). ER on the incompatible current trial that followed compatible preceding trial was lower in the Incompatible block than in the Neutral block (*p* < 0.001). Finally, ER was lower on the compatible current trial than on the incompatible current trial in all cases of the block type × the preceding trial type; Neutral block × Compatible preceding trial, *p* < 0.001; Neutral block × Incompatible preceding trial, *p* < 0.001; Incompatible block × Compatible preceding trial, *p* = 0.003; Incompatible block × Incompatible preceding trial, *p* = 0.005.

### BLOCK-WISE ADAPTATION EFFECT

**FIGURE 2:**
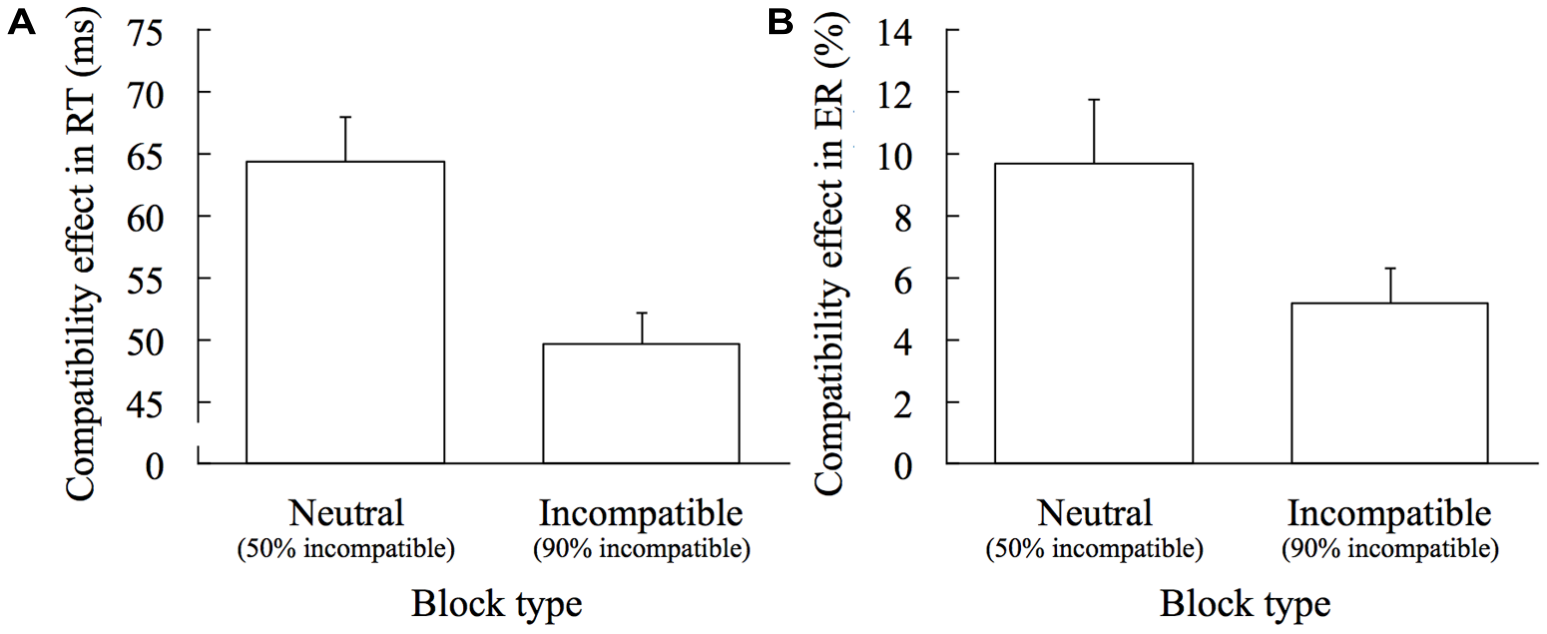
**Block-wise adaptation effect.** Error bars show 1 SEM. **(A)** Comparing the compatibility effects in RT (RT on incompatible trials minus RT on compatible trials) between the Neutral and the Incompatible blocks. **(B)** Comparing the compatibility effects in ER (ER on incompatible trials minus ER on compatible trials) between the Neutral and the Incompatible blocks.

The compatibility effects in RT (RT on the incompatible trials minus RT on the compatible trials) and ER (ER on the incompatible trials minus ER on the compatible trials) in the Neutral and the Incompatible blocks are shown in **Figure 2**. There was a significant difference in the compatibility effects, both in RT and ER, between the Neutral and the Incompatible blocks, *t*(19) = 5.482, *p* < 0.001, *d* = 1.772 (**Figure 2A**) and *t*(19) = 3.139, *p* = 0.005, *d* = 0.629 (**Figure 2B**), respectively. An adaptation index, which is calculated by subtracting the compatibility effect in the Incompatible block from that in the Neutral block, was 15.00 ms in RT and 4.51% in ER.

### TRIAL-BY-TRIAL ADAPTATION EFFECT

**FIGURE 3:**
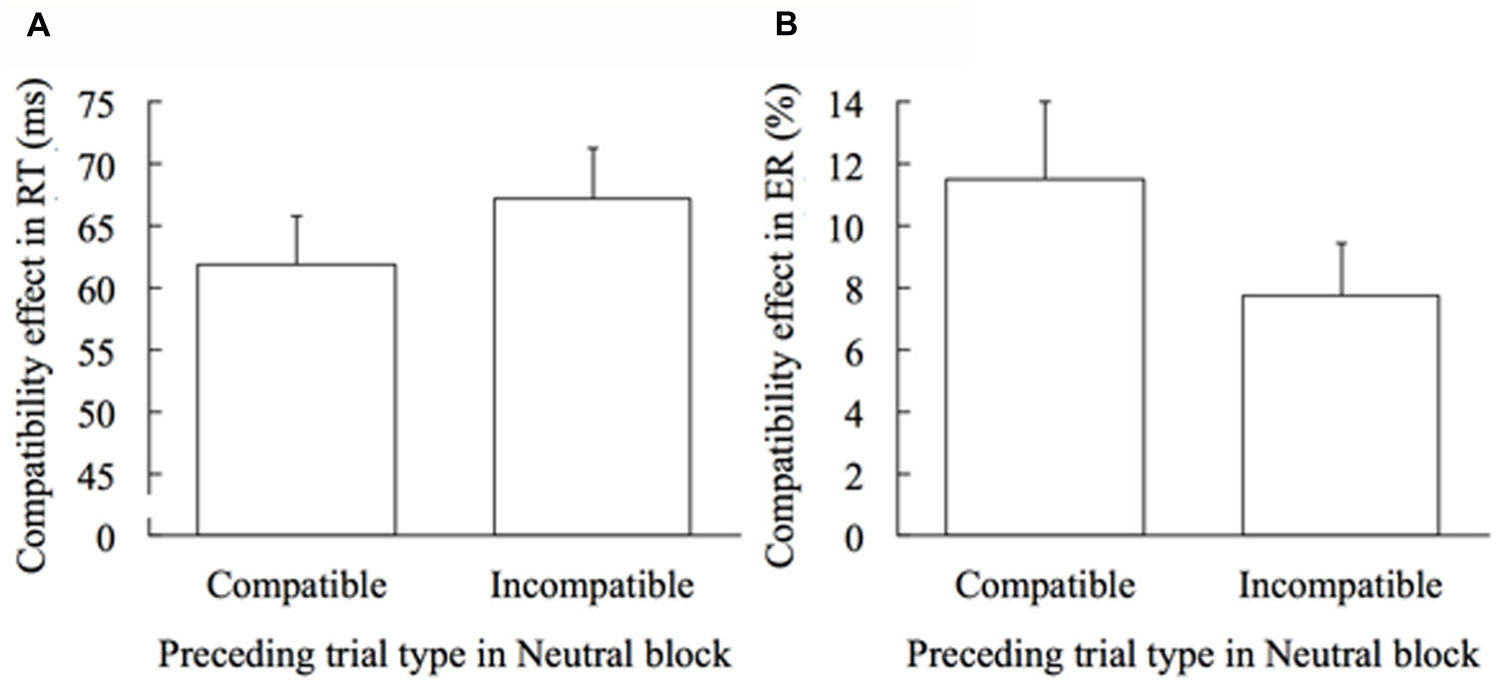
**Trial-by-trial adaptation effect in the Neutral block.** Error bars show 1 SEM. **(A)** Effect of the preceding trial type on the compatibility effect in RT. **(B)** Effect of the preceding trial type on the compatibility effect in ER.

Next, to examine the trial-by-trial adaptation, the compatibility effect in RT and ER was calculated separately for trials immediately preceded by the compatible trial and for trials immediately preceded by the incompatible trial in the Neutral block. **Figure 3** shows the effect of the preceding trial type (compatible or incompatible) on the compatibility effects in the Neutral block. The compatibility effect in RT was larger with marginal significance when the preceding trial type was incompatible compared to compatible, *t*(19) = 1.860, *p* = 0.079, *d* = 0.299 (**Figure 3A**). Contrarily, the compatibility effect in ER was significantly smaller when the preceding trial type was incompatible than when it was compatible, *t*(19) = 3.253, *p* = 0.004, *d* = 0.402 (**Figure 3B**). RT adaptation was -5.28 ms, and ER adaptation was 3.74%.

## DISCUSSION

In the present study, we examined the role of visual awareness for the block-wise and trial-by-trial adaptations. Block-wise adaptation was clearly observed; the compatibility effect was smaller in the Incompatible block than in the Neutral block, in both the RT and the ER measures. In addition, the result of the prime discrimination task showed that participants could not discriminate the pointing direction of the prime and the post-task interview revealed that no participants noticed any difference between the blocks, ensuring that the effect of block-wise adaptation on the task performance was derived unconsciously.

These results may give an empirical support to the contingency learning model for the block-wise adaptation, because it has been shown that the contingency learning of proportion congruency can be achieved without awareness (Schmidt et al., 2007). Furthermore, our result is consistent with the evidence of sequence-specific learning effect, which shows that compatibility effect decreases gradually when the order of stimulus-presentation is determined with sequential regularity, though participants did not notice such a regularity (Deroost et al., 2012). These findings, together with our result, suggest that adaptation for the long-term conflict context should progress in an implicit manner without participant’s awareness of the conflict

Looking from another angle, the present result would be explained by the Adaptation to the Statistics of the Environment (ASE) model (Kinoshita et al., 2008, 2011), which argues that both the trial-by-trial sequential effect and the proportion effect are driven by the history of trial difficulty. Recent study showed that the conflict awareness could be developed even when visual awareness is absent, and this conflict awareness triggered the conflict adaptation in the masked priming task (Desender et al., 2014). In the present experiment, participants might have felt stronger difficulty in the Incompatible block than in the Neutral block, thereby leading to the conflict awareness. However, although the ASE model is proposed to account for both the trial-by-trial and block-wise effects, the trial-by-trial RT adaptation was not observed in the present results. This suggests that the awareness of stimulus incompatibility between the task-relevant information and the task-irrelevant information is necessary to generate trial-by-trial RT adaptation.

On the other hand, ER data showed that the compatibility effect was changed not only by the block-wise but also the trial-by-trial context. Although some researchers denied the trial-by-trial adaptation in the masked priming task (Kunde, 2003; Ansorge et al., 2010), the present result showed that the trial-by-trial context caused certain change. Nevertheless, because the compatibility effect is likely to be manifested in both RT and ER data, it may be the case that the speed/accuracy trade-off (e.g., post-error slowing) is distinguished from the conflict adaptation (Notebaert and Verguts, 2011). As previously noted, Francken et al. (2011) demonstrated the unconscious trial-by-trial effect only on the accuracy measure (**Table 1**). Furthermore, the results of accuracy reported by Kunde (2003) appear to show a difference between conditions with compatible and incompatible preceding trials, but only through visual inspection of the graph, as the statistical analysis of the data was not provided. Taken together, these results may suggest that the trial-by-trial effect is limited to ER adaptation and does not cause RT adaptation; rather, it may cause an increase of the compatibility effect in RT as in the case of the present result (**Figure 3A**), probably due to speed/accuracy trade-off. Note that the results of other masked priming studies support this view, that is, the trial-by-trial effect on responders’ cautiousness has been shown in the masked Go/No-Go task (van Gaal et al., 2008) and in the stop-signal task (van Gaal et al., 2009). These tasks do not require response selection; thus, speed/accuracy trade-off would be sufficient to improve the performance. However, this interpretation needs to be validated by more evidences in the future research.

In summary, the present study investigated the role of awareness in the trial-by-trial and block-wise adaptation to the response conflict. A partial trial-by-trial adaptation (speed/accuracy trade-off) and complete block-wise adaptation (enhancement in both speed and accuracy) were found in the masked priming task. Therefore, we can conclude that the stimulus awareness is not necessary for the block-wise adaptation. The sustained conflict context boosts the conflict resolution even unconsciously. On the other hand, when a response conflict was experienced unconsciously in the preceding trial, the process of conflict resolution would not be completely facilitated, triggering only speed/accuracy trade-off.

## Conflict of Interest Statement

The authors declare that the research was conducted in the absence of any commercial or financial relationships that could be construed as a potential conflict of interest.
